# The Preliminary Education of a Medical Man

**Published:** 1910-09-03

**Authors:** 


### THE PRELIMINARY EDUCATION OF A MEDICAL MAN.

The career of a medical man includes so many and such varied activities—of body as well as of mind—and calls for the exercise of such numerous attributes and such development of character, that the preliminary education of the complete physician and surgeon should properly be a liberal one in the very fullest sense of that somewhat elastic term. Unfortunately, in this respect, the professional education of the medical student *in statu pupillari* is so prolonged and exacting that he who seeks to qualify in reasonable time and to make the most of his opportunities of fitting himself for his subsequent career, has but little time or chance for the cultivation of the classics or the fine arts. Such general education as he can get is, in most cases, only to be obtained before his professional education begins; though there will no doubt always be brilliant exceptions to be quoted against this generalisation.

Lest this dogmatism evoke indignant protest, let it be admitted that it holds good only for the average man, not for those exceptionally talented folks, of whom a handful are always to be found in any society, who seem able to achieve a mastery over difficult subjects with a facility such as the multitude can only envy without being able to copy. Only recently, for example, the death of Sir Seymour Haden reminded the medical profession of one of its distinguished members whose eminence as an artist was certainly as great as (if not greater than) the marked success which he achieved in practice. There are also still two or three well-known consulting physicians in England who are respected almost as much for their scholarly qualities as for their professional distinctions. But yet it remains true that for the bulk of medical men in practice the commencement of their medical studies sees the end of all other academic education.

### The Essentials of a Liberal Education.

However, a liberal education includes much that no pedagogue can teach, and much that is not found within the covers of lexicons or algebra books. For one thing, the introductory studies of the first two or three years of the curriculum include subjects some acquaintance with which every educated man with pretensions to culture should attain to. Elementary biology, chemistry, and physics, as required by the examining boards for the "preliminary scientific" examinations do not demand very profound knowledge; but they open the doors of a most fascinating treasure-house, and dull must that student be who does not enter it for a short time. He who can study the excellently-chosen types of invertebrate and vertebrate zoology of this syllabus without feeling and gratifying some desire for further glimpses of the fascinating subject of comparative anatomy; he who can "get through" his examination in biology without the impulse to read the "Origin of Species" or the works of Haeckel and Huxley: these are men of so dull a spirit that they are not likely to make any great mark as doctors; and, in fact, are not of the clay that the real doctor is made of. As a direct preparation for the study of the medical sciences strictly so called, these subjects claim an unduly large share of the five years allotted (theoretically) to the medical curriculum; as an exercise for the development of the scientific faculties they are so useful that few of those who regard the full mental equipment of the practitioner would see them cut down. The medical profession is essentially scientific; and if every doctor cannot be imbued with the true scientific spirit, at least he can be taught to think in a scientific way. Any scheme of education which will promote this end is a worthy one.

### The Question of a Classical Education.

All that, perhaps, is not strictly preliminary to medical study, but rather an integral part of it. What may profitably be considered is the best form of general education for the future medical man; and this resolves itself mainly into the question of what form of school education is most desirable, though there is another aspect which shall be dealt with presently. At the British public schools the study of the classics is slowly but surely losing the predominance which it once had. Whether or not this is matter for congratulation or the reverse is a debatable subject; at least it is agreed that any training which is satisfactorily to replace the old-fashioned Greek and Latin must be no mere haphazard and half-hearted scraping acquaintance with a few problems in geometry and the laws which govern the expansion of gases. Probably the majority of present-day medical men would say that as a deliberate preparation for medical study Greek and Latin are of very little use indeed. German, French, and, above all, English, with a thorough mathematical training, and a little simple chemistry and physics; all this constitutes a curriculum amply sufficient to occupy all the working hours of any boy up to the age of sixteen or seventeen. It is not to be denied that many men who are ornaments to the profession have been brought up on little else but Greek and Latin in the traditional fashion up to eighteen or nineteen; and it is true that some of them are convinced that their education has greatly benefited them and helped them in their medical studies. But for the vast majority, to whom the dead languages are a distasteful bugbear, such an education is too often a waste of valuable time and money. At the public schools, it is true, the modern side is in many cases a refuge for the idle or the stupid, for whom the headmaster sees little prospect of distinction on the classical side; but this state of things is slowly altering for the better, and there are some schools at which, as a general training for the mind, the modern side leaves little to be desired. It is safe to add that very, very seldom does the study of English literature, language, composition, history, and economics receive anything like the share of attention which is its due; and the spectacle of University graduates, really clever men and eminent in the medical profession, unable to write three consecutive lines without a spelling mistake, is one that is not only painful, but unfortunately far from rare.

### Sport and Athletics.

However, it is not only to the classroom that the education of the British public-school boy is confined. The school games, the discipline of school life, the "tone" of the house, and other factors are very important parts of that system which beyond doubt has left its imprint indelibly on the manhood of the nation. To learn the lessons of manliness, modesty, self-reliance, honour, which the right sort of boy picks up at a good public school, is a preparation for a medical career second to nothing, neither to classical nor modern academic study. That the cult of athletics is overdone at many of our best-known schools is an accusation in which there is probably more than a grain of truth; but on the other hand there is no doubt that too much athletics is better than none at all. For medical men in particular it is vastly important that an iron constitution and a vigorous frame should be built up in youth; for practice is an arduous and exacting pursuit which demands all the health and strength that a man can bring to it. The medical profession is no place for a weakling, mentally or physically; and no education, preliminary or otherwise, will fit a man for general practice if he be constitutionally sound. With a few rare and notable exceptions the teaching staff at the medical schools take little interest in the students' games; but such an indifference is most regrettable, inasmuch as the discipline of English sports is in reality quite as much a part of English education as is the curriculum of instruction.

### Other Assets.

Even over and beyond the education which a boy gets at school, the environment of his home life, his hobbies and recreations, his general tastes, too, will all make a difference to the final product. Thus many a country-bred lad revolts at the idea of spending his days toiling in some great but grimy city, and cheerfully sacrifices a wider reputation or a larger income in exchange for the delights of country life such as can be got in a country practice. Again, many a flourishing country practice has been founded on the sporting instincts and tastes of its possessor. A house-surgeon at a country hospital who can throw a fly skilfully, bring down his bird with certainty, keep in the first flight after hounds, or make his century for the local cricket club—such an all-round sportsman if he be of a genial nature makes a host of friends and is as likely as not to be pressed by some of them to put up his plate in the district, or to be taken into partnership on favourable terms by one of the established practitioners. The capacity for making friends in this way is an asset which has been shown over and over again by experience to be no contemptible one; and even in a big town the record of an athletic career will often help the young medical man in many ways. Then, again, some interesting hobby is almost a necessity for the practising doctor. In these pages practitioners have from time to time described their own particular favourite ways of obtaining that relaxation which is so valuable to the hardworked and often overworked doctor. In many cases these hobbies import a close study, a culture in fact, which is really a component part of a broad-minded man's education. Not many months ago the daily Press announced the sale by a well-known surgeon of five Chinese vases at ten thousand pounds apiece. It is safe to say that this gentleman had given no such price for them originally; but having cultivated the taste of a connoisseur in such pottery he had become an expert and had been able to invest his money in a form of property beside which rubber plantations are comparatively insignificant.

### The All-round Man.

In short, it is the all-round man who gets on best in the medical profession, no matter what branch of it is concerned. The man who knows his work thoroughly and practically, yet is no mere bookworm or recluse: the man who is gentle of hand and sympathetic of manner, yet has the strength requisite for the harassing life of the practitioner, never off duty and never spared: the man who is not merely a theorist, but an experienced man of the world; such is the man who succeeds in the medical profession. The man thus sketched is, of course, to a large extent born, not made; yet education and environment have an even greater share in shaping character, and no care and trouble are thrown away which shall insure that a man (or for that matter a woman) intended for that profession shall have these various attributes developed to the highest degree which can in the circumstances of each particular case be reasonably expected.

